# Prognostic value of Prognostic Nutritional Index in cancer: an umbrella review of systematic review and meta-analysis

**DOI:** 10.3389/fnut.2025.1706534

**Published:** 2026-01-21

**Authors:** Jiyu Yang, Cuixue Wang, Jian Xue, Xiao Ma, Yingze Hou, Haoran Yuan, Kaiyan Xia, Long Piao, Xianchun Zhou

**Affiliations:** 1 Central Laboratory, The Affiliated Hospital of Yanbian University, Yanji, China; 2 Department of Oncology, Yanbian Hospital Affiliated to Yanbian University, Yanji, China; 3 Department of Nephrology, Siping Central People's Hospital, Siping, China; 4 Department of Oncology, Xiangshui County People's Hospital, Yancheng, China; 5 Department of Rehabilitation Medicine, Yanbian Hospital Affiliated to Yanbian University, Yanji, China

**Keywords:** cancer, outcomes, prognostic, Prognostic Nutritional Index, umbrella review

## Abstract

**Background:**

The Prognostic Nutritional Index (PNI) is a critical biomarker reflecting the nutritional and immune status of cancer patients. Its association with the prognosis of various malignant tumors has been extensively investigated. However, a comprehensive systematic evaluation of the prognostic significance of PNI across different cancer types remains limited.

**Methods:**

This umbrella review conducted a systematic search of PubMed, Embase, Web of Science, and the Cochrane Library to include meta-analyses that assessed survival outcomes related to cancer across diverse malignancy categories. The quality of the evidence was evaluated utilizing the GRADE and AMSTAR tools. Effect sizes, expressed as hazard ratios (HR) or odds ratios (OR), were synthesized using either random-effects or fixed-effects models. Additionally, analyses of heterogeneity, indicated by I^2^, and assessments of publication bias were performed as appropriate.

**Results:**

The Prognostic Nutritional Index (PNI) demonstrates associations across multiple cancer types, with a low PNI significantly linked to poor prognosis in 12 categories of malignant tumors. The HRs for 103 outcomes ranged from 1.15 to 2.98 (*p* < 0.05). The overall quality of evidence is predominantly low or very low, as assessed by the GRADE criteria, affecting 89.3% of outcomes. This is largely attributed to heterogeneity (47.8% with I^2^ > 50%), publication bias (40%), and limitations in AMSTAR scoring. Notably, 11 outcomes provided moderate-quality evidence, all indicating substantial effect sizes. Specifically, PNI shows significant prognostic value in breast cancer (preoperative overall survival [OS] HR = 2.56, postoperative HR = 2.63), cervical cancer (OS HR = 2.98), and among patients receiving immunotherapy (e.g., lung cancer immune checkpoint inhibitor [ICI] group OS HR = 2.68).

**Conclusion:**

A low Prognostic Nutritional Index is consistently associated with poorer outcomes across various cancer types, particularly in predicting responses to immunotherapy. However, due to the current limited quality of evidence, it cannot yet be recommended as a standalone prognostic or decision-making tool across all tumors. Future rigorously designed, multi-center studies are required to validate its clinical utility.

**Systematic review registration:**

https://www.crd.york.ac.uk/PROSPERO/,CRD42025111093.

## Introduction

Malnutrition is a prevalent comorbidity in cancer patients, forming a vicious cycle with inflammation and immune exhaustion that impairs treatment tolerance, quality of life, and prognosis (1–5). This cycle is driven by tumor-induced nutrient consumption, pro-inflammatory cytokine secretion, and subsequent immune dysfunction—key pathways linking nutritional status to cancer outcomes (6–8). Accumulating evidence confirms nutritional status as an independent prognostic factor for cancer survival (9, 10). The Prognostic Nutritional Index (PNI), introduced by Onodera et al. (11) in 1984, integrated serum albumin and peripheral lymphocyte counts to reflect both nutritional reserves and immune status. Extensive research has validated PNI as a prognostic marker across malignancies: low preoperative PNI predicts poor survival in hepatocellular carcinoma (12, 13), prostate cancer (14, 15), biliary tract cancers (16), and gastric cancer—where it also modulates immunotherapy efficacy (6–8).

Despite numerous meta-analyses investigating the prognostic role of PNI, critical gaps remain. Existing studies are fragmented across tumor types, with inconsistent evidence quality, unaddressed heterogeneity, and potential publication bias. No high-level synthesis has systematically evaluated the prognostic consistency, evidence reliability, or applicability of PNI across diverse malignancies. This lack of comprehensive integration hinders clinicians from translating PNI findings into practice. To address this, we conducted an umbrella review to synthesize existing meta-analyses, clarify the prognostic utility of PNI across cancer types, and provide clinicians with rigorous, evidence-based guidance.

## Methods

### Umbrella review methods

We conducted a systematic identification, extraction, and analysis of data from published systematic reviews and meta-analyses that investigated the associations between the Prognostic Nutritional Index (PNI) and survival outcomes in cancer patients. The PNI was calculated using the formula: serum albumin (g/dL) + 5 × total lymphocyte count (10^9^/L), with equivalent unit conversions applied as necessary. Pooled statistical results were obtained from meta-analyses. Consequently, systematic reviews that did not include meta-analyses were excluded from this umbrella review. This study is prospectively registered with PROSPERO (CRD42025111093) (https://www.crd.york.ac.uk/PROSPERO/).

### Literature search methodology

We conducted a comprehensive search of PubMed, Embase, Web of Science, and the Cochrane Database of Systematic Reviews to identify systematic reviews and meta-analyses published from the inception of each database until April 2025 (the date of the last update). Utilizing the Scottish Intercollegiate Guidelines Network (SIGN) criteria, we employed a search strategy that combined Medical Subject Headings (MeSH), keywords, and text terms as follows: (((“Neoplasms”[Mesh]) OR ((((((((((((Tumors) OR (Neoplasia)) OR (Neoplasias)) OR (Neoplasm)) OR (Tumor)) OR (Cancer)) OR (Cancers)) OR (Malignant Neoplasm)) OR (Malignancy)) OR (Malignancies)) OR (Malignant Neoplasms)) OR (Carcinoma))) AND ((prognostic nutritional index) OR (PNI))) AND (systematic review OR meta-analysis). Two investigators (YJY and WCX) independently screened the titles and abstracts and evaluated the full texts for eligibility. Any disagreements were resolved by consultation with a third reviewer (ZXC). Additionally, we manually examined the reference lists of the included studies to identify further relevant meta-analyses.

### Inclusion criteria

All included meta-analyses were categorized based on the cancer type, specific patient subgroup (e.g., those receiving immunotherapy or surgery), and outcome indicators (e.g., OS and PFS). For overlapping meta-analyses addressing the same patient population and outcome, a predefined hierarchical criteria was applied to select only one “best” meta-analysis for inclusion in this umbrella review:

1. Prioritizing timeliness: If the publication time interval between meta-analyses was ≥ 2 years, the most recently published study was included.
2. Prioritizing sample size: If the publication time interval was < 2 years, the study with the larger total sample size was included.
3. Prioritizing methodological quality: If the above criteria could not differentiate, the study with a higher AMSTAR score was selected.

A limitation of our approach is that potential patient cohort overlap within different subgroup analyses of the same meta-analysis (e.g., the overall population analysis and one of its subgroups) was not identified or adjusted for.

### Quality assessment of methods and evidence

We employed the AMSTAR (A MeaSurement Tool to Assess systematic Reviews) tool to evaluate the methodological quality of the included meta-analyses (17). This instrument provides a quantitative scoring system ranging from 0 to 11 points based on 11 predefined items. Each item was assessed as “yes” (scoring 1 point), “no,” “unable to answer,” or “not mentioned” (the latter three responses scoring 0 points). Based on consensus, we established the following quality thresholds: studies scoring ≥ 8 points were classified as high quality (and accordingly rated as “no serious risk of bias” in the GRADE assessment), those scoring 4–7 points as moderate quality, and those scoring ≤3 points as low quality.

### Data analysis

The pooled effect sizes (e.g., HR and OR) and their 95% confidence intervals reported in this umbrella review were directly extracted from the pooled results of the included original meta-analyses. Specifically, we adopted the statistical model (fixed-effects or random-effects model) selected by the authors of the original meta-analyses based on their heterogeneity test results. We performed GRADE evidence grading by integrating the heterogeneity (quantified by the I^2^ statistic) and publication bias (assessed via Egger’s regression test) of each included study.

## Results

**Figure 1:**
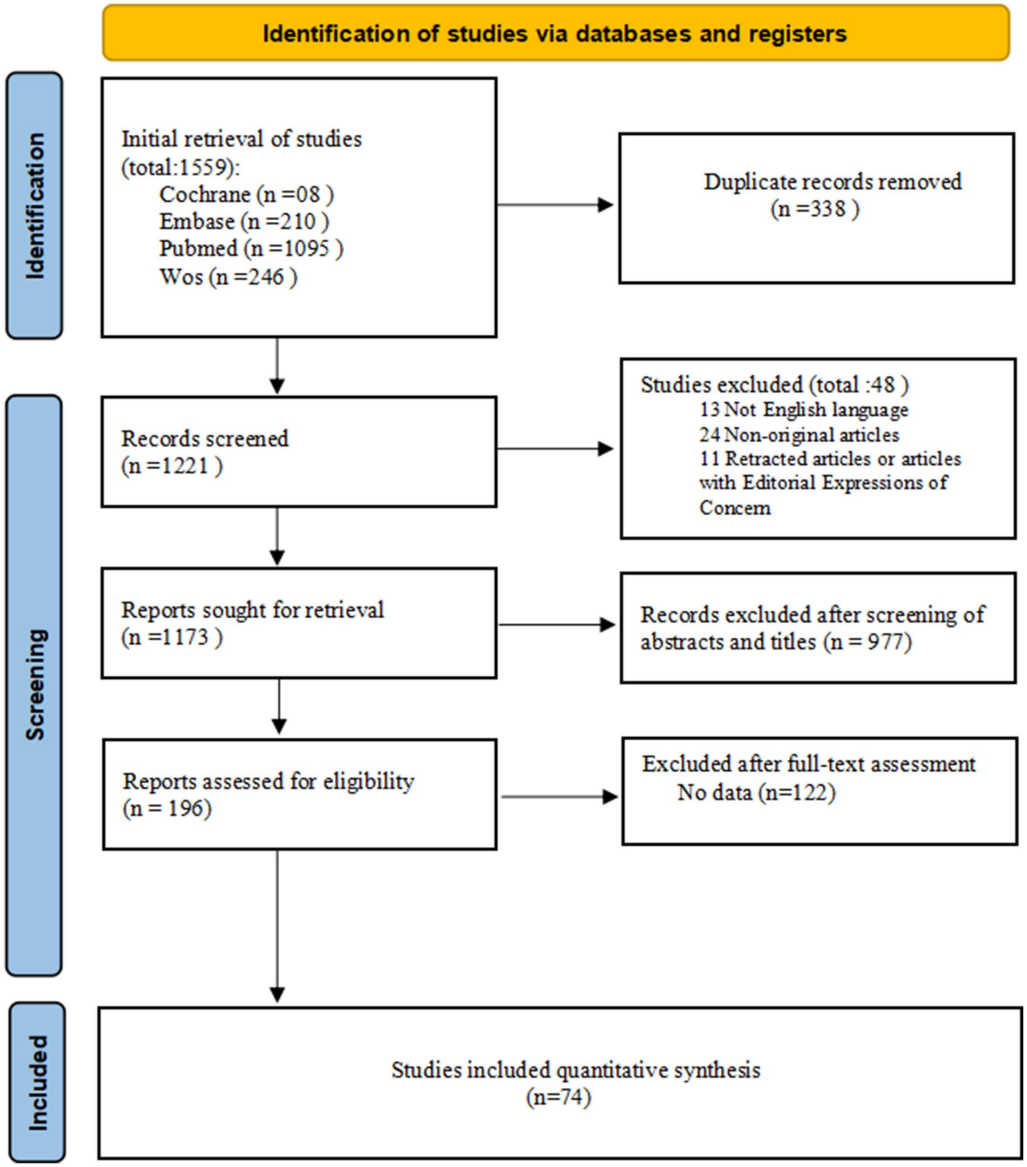
Flowchart depicting the literature search and study selection process for this umbrella review. Initial retrieval from Cochrane (8), Embase (210), PubMed (1095), and WoS (246) yielded 1559 records. After removing 338 duplicates, 1221 records were screened, with 48 excluded (13 non-English, 24 non-original, 11 retracted). Among 1173 sought records, 977 were excluded after screening, 122 after full-text assessment of 196 reports, and 74 studies were finally included in quantitative synthesis.

Figure 1 presents a flowchart detailing the literature search and selection process. Through a systematic literature search, 1,221 unique articles were initially identified. Upon applying our inclusion criteria, 74 meta-analyses were selected, comprising 159 outcome measures, from which 111 outcome indicators were extracted. The detailed AMSTAR scoring for each study is provided in Supplementary Table S1. These meta-analyses pertain to malignant tumors across various systems, including the digestive, reproductive, urinary, and respiratory systems, as well as head and neck cancers, lymphomas, and other malignancies not specifically classified (Supplementary Table S2).

**Figure 2:**
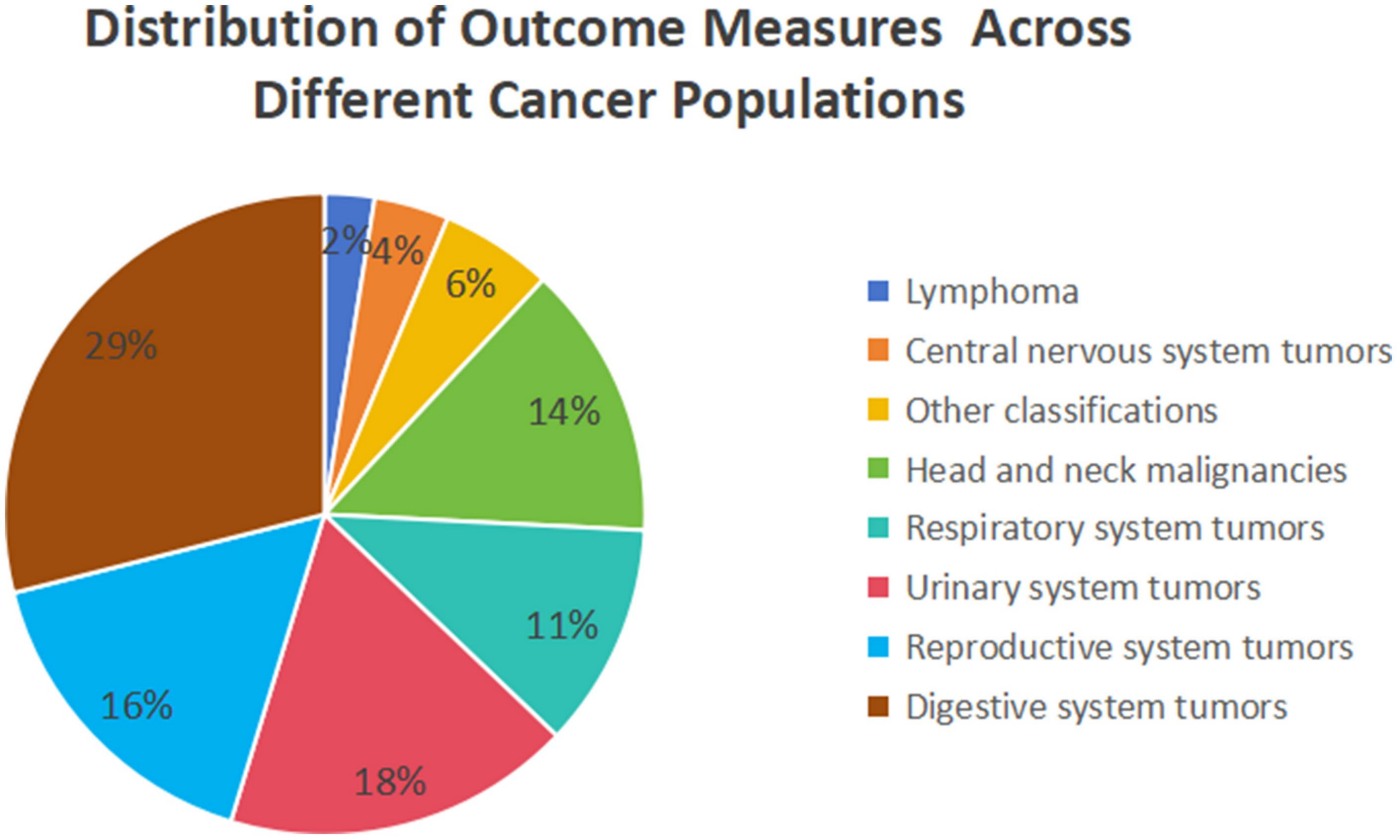
Pie chart showing the distribution of outcome measures across different cancer populations. Digestive system tumors account for the largest proportion (29%), followed by urinary system tumors (18%), reproductive system tumors (16%), head and neck malignancies (14%), respiratory system tumors (11%), other classifications (6%), central nervous system tumors (4%), and lymphoma (2%).

**Figure 3:**
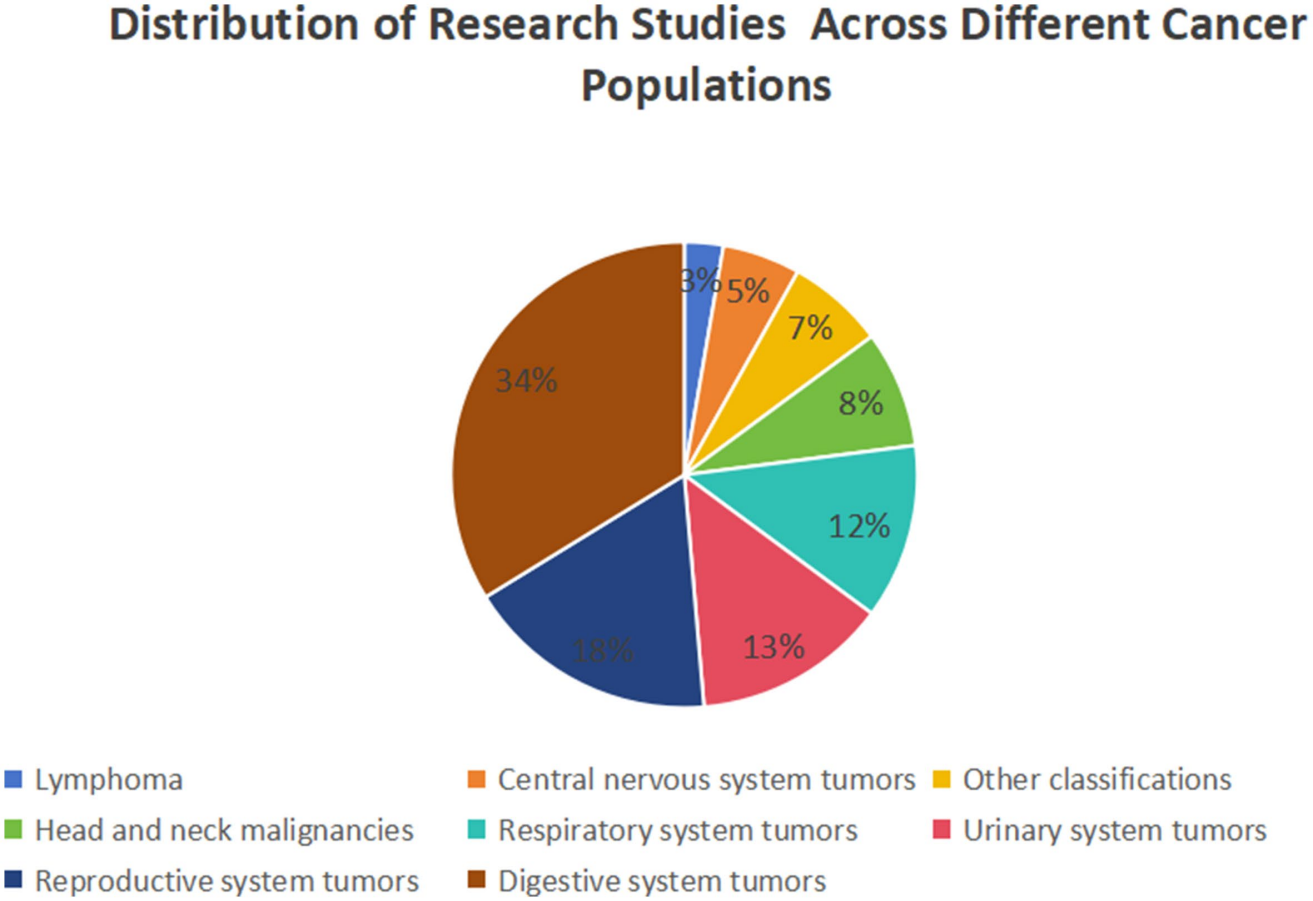
Pie chart illustrating the distribution of research studies across various cancer populations. Reproductive system tumors have the highest share (34%), followed by lymphoma (18%), urinary system tumors (13%), respiratory system tumors (12%), head and neck malignancies (8%), central nervous system tumors (7%), other classifications (5%), and digestive system tumors (3%).

The majority of included meta-analyses investigate the relationship between Prognostic Nutritional Index (PNI) and gastrointestinal tumors (*n* = 25), followed by reproductive (*n* = 13), urological (*n* = 10), and respiratory (*n* = 9) system tumors in relation to PNI. The studies investigating the relationship between lymphoma and the Prognostic Nutritional Index (PNI) are notably limited in number (*n* = 2). All associations suggest a negative correlation with PNI, with 103 instances reaching statistical significance. Upon evaluating the quality of evidence using the GRADE framework, the majority of the 103 significant outcomes were deemed to be of “low” or “very low” quality (*n* = 34 and *n* = 58, respectively). The remaining 11 outcomes were classified as “moderate” quality. Notably, no evidence was rated as “high” quality in this review (Supplementary Tables S2, S3). Supplementary Table S3 presents the final GRADE ratings for all assessed associations, along with detailed justifications for each downgrading decision organized by GRADE domain. Specific reasons for rating adjustments include: Risk of Bias (e.g., evaluated through AMSTAR scores), Inconsistency (e.g., I^2^ > 50%), Imprecision (e.g., wide confidence intervals), and Publication Bias (e.g., assessed via funnel plot inspection or Egger’s test). Figures 2, 3 depict the distribution of studies and outcomes across various cancer populations.

### Overall summary of findings

A core finding of this umbrella review is that low PNI is significantly associated with poor prognosis across 12 categories of malignant tumors, encompassing the digestive, reproductive, urinary, respiratory, head and neck, lymphoid, and central nervous system malignancies, as well as unclassified tumors and elderly cancer patient populations. Among the 111 extracted outcome indicators, 103 (92.8%) showed statistically significant negative correlations between PNI and prognosis, with HR values ranging from 1.15 to 2.98 (*p* < 0.05), confirming PNI’s stable prognostic predictive capacity across diverse cancer types.

### Outcomes of digestive system cancer

#### In the context of pancreatic cancer and periampullary cancer following post-pancreatoduodenectomy

**Figure 4:**
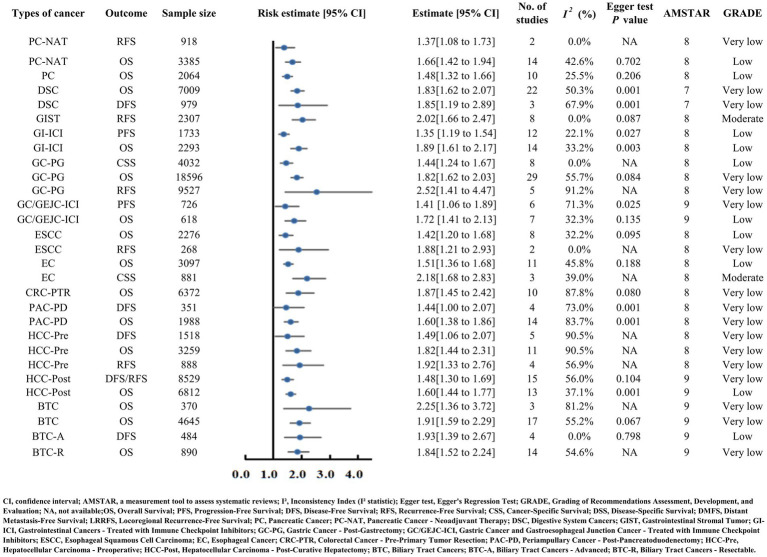
Forest plot presenting risk estimates and 95% confidence intervals for digestive system cancers (pancreatic, gastric, colorectal, hepatocellular carcinoma, etc.) and related outcomes (OS, PFS, DFS, CSS). It includes key metrics such as sample size, number of studies, heterogeneity (I²), Egger test P values, AMSTAR scores, and GRADE evidence quality (ranging from very low to moderate).

Pancreatic cancer, periampullary cancer post-pancreatoduodenectomy, and pancreatic cancer receiving neoadjuvant therapy (PC-NAT) all correlated with poorer prognosis in the setting of reduced PNI (pancreatic cancer OS HR = 1.48; periampullary cancer OS HR = 1.60, DFS HR = 1.44; PC-NAT OS HR = 1.66, RFS HR = 1.37) (Supplementary Table S2) (18–20). Both analyses were assigned a very low GRADE quality rating (Figure 4).

#### Esophageal, gastric, and gastroesophageal junction cancers

Esophageal carcinoma (EC) and esophageal squamous cell carcinoma (ESCC) exhibited comparable OS risk estimates with decreased PNI (EC HR = 1.51, ESCC HR = 1.42) (Supplementary Table S2) (21, 22). A notable moderate-quality finding emerged for EC: patients with low PNI demonstrated significantly poorer cancer-specific survival (CSS HR = 2.18), supported by consistent results across three studies with minimal heterogeneity (I^2^ = 39.0%) (Figure 4) (23).

Patients with gastric cancer post-gastrectomy (GC-PG) and those with gastric or gastroesophageal junction carcinoma receiving immune checkpoint inhibitors (GC/GEJC-ICI) experienced adverse survival outcomes with reduced PNI (GC-PG OS HR = 1.82, RFS HR = 2.52; GC/GEJC-ICI OS HR = 1.72, PFS HR = 1.41) (Supplementary Table S2; Figure 4) (24, 25).

#### Colorectal, liver, and biliary tract cancers

Reduced PNI was associated with compromised survival in colorectal carcinoma with preoperative tumor resection (CRC-PTR, OS HR = 1.87), hepatocellular carcinoma (HCC-Pre OS HR = 1.82, HCC-Post OS HR = 1.60, DFS/RFS HR = 1.48), and biliary tract carcinoma (BTC, advanced OS HR = 2.25, resectable OS HR = 1.84, comprehensive OS HR = 1.91, DFS HR = 1.93) (Supplementary Table S2; Figure 4) (16, 26–28).

#### Other categories

Patients with overall digestive system carcinoma (DSC) faced poorer OS with low PNI (HR = 1.83) (Supplementary Table S2) (29). Gastrointestinal stromal tumor (GIST) yielded moderate-quality evidence: reduced PNI strongly correlated with elevated recurrence-free survival (RFS) risk (HR = 2.02), based on eight studies with no heterogeneity (I^2^ = 0.0%) and no significant publication bias (Egger’s test *p* = 0.001) (30). Gastrointestinal cancers treated with immune checkpoint inhibitors (GI-ICI) also demonstrated worse PFS (HR = 1.35) and OS (HR = 1.89) in the context of low PNI (Supplementary Table S2; Figure 4) (31).

### Outcomes of reproductive system cancers

#### Breast cancer

**Figure 5:**
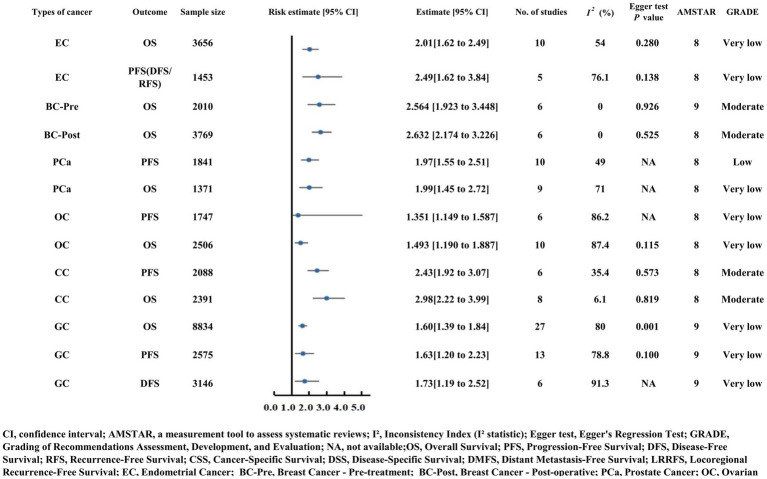
Forest plot displaying risk estimates and 95% confidence intervals for reproductive system cancers (breast, cervical, ovarian, endometrial carcinoma) and outcomes including OS and PFS. Sample sizes range from 1371 to 8834, with additional details on study count, heterogeneity, Egger test results, AMSTAR scores, and GRADE quality (very low to moderate).

Moderate-quality evidence confirmed that PNI strongly predicts OS in breast cancer patients, both preoperatively and postoperatively. Preoperative patients (*n* = 2,010) exhibited an OS HR of 2.56, while postoperative patients (*n* = 3,769) had an OS HR of 2.63. Both subgroups showed no heterogeneity (I^2^ = 0.0%) and robust methodological quality (AMSTAR score = 9), with no evidence of publication bias (Egger’s test *p* = 0.926 for preoperative, *p* = 0.525 for postoperative) (Figure 5) (32, 33).

#### Prostate cancer

Prostate cancer patients with reduced PNI faced higher risks of diminished progression-free survival (PFS HR = 1.97) and overall survival (OS HR = 1.99) (Supplementary Table S2; Figure 5) (34).

#### Ovarian cancer (OCa)

For ovarian cancer (OCa), a low PNI is significantly associated with poorer PFS (HR = 1.351) and OS (HR = 1.493) (Supplementary Table S2; Figure 5) (35).

#### Endometrial carcinoma (EC) and cervical carcinoma (CC)

Endometrial cancer (EC) patients demonstrated poorer OS (HR = 2.01) and PFS/DFS/RFS (HR = 2.49) with low PNI (Supplementary Table S2; Figure 5) (36, 37).

Cervical cancer (CC) provided moderate-quality evidence for the strong prognostic value of PNI: PFS HR = 2.43 (six studies, *n* = 2,088) and OS HR = 2.98 (eight studies, *n* = 2,391). Heterogeneity was low (I^2^ = 35.4% for PFS, 6.1% for OS), and no publication bias was detected (Egger’s test *p* = 0.573 for PFS, *p* = 0.819 for OS), with an AMSTAR score of 8 indicating high methodological rigor (Figure 5) (38).

#### Overall gynecological cancers (GC)

Across gynecological cancer studies, low PNI correlated with poorer OS (HR = 1.60), PFS (HR = 1.63), and DFS (HR = 1.73) (Supplementary Table S2; Figure 5) (39).

### Outcomes of urinary system cancers

#### Renal cell carcinoma (RCC)

**Figure 6:**
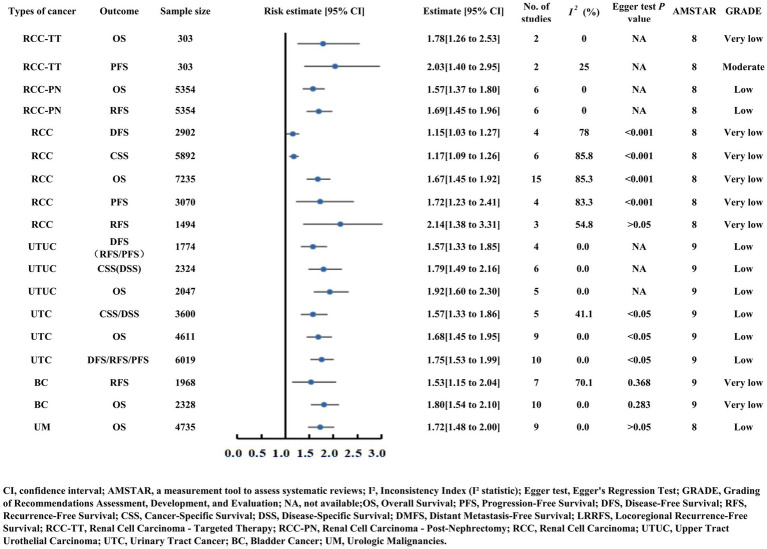
Forest plot of risk estimates and 95% confidence intervals for urinary system cancers (renal cell carcinoma, upper tract urothelial carcinoma, bladder cancer, uveal melanoma) and related outcomes (OS, PFS, RFS, CSS). The plot includes sample size, number of studies, I² heterogeneity, Egger test P values, AMSTAR scores, and GRADE levels.

Renal cell carcinoma (RCC) patients who underwent nephrectomy (RCC-PN, OS HR = 1.57, RFS HR = 1.69) and those receiving targeted therapy (RCC-TT, OS HR = 1.78) demonstrated worse survival with low PNI (Supplementary Table S2; Figure 6) (40). Moderate-quality evidence emerged for RCC-TT patients: low PNI was linked to shorter PFS (HR = 2.03) based on two consistent studies (*n* = 303) (Figure 6). In broader RCC analyses, OS (HR = 1.67), PFS (HR = 1.72), RFS (HR = 2.14), CSS (HR = 1.17), and DFS (HR = 1.15) (Supplementary Table S2; Figure 6) (41).

#### Upper tract urothelial carcinoma (UTUC) and urinary tract cancer (UTC)

Upper tract urothelial carcinoma (UTUC) patients with low PNI demonstrated poorer OS (HR = 1.92), CSS/DSS (HR = 1.79), and DFS/PFS/RFS (HR = 1.57). Urothelial carcinoma (UTC) patients showed similar trends with OS (HR = 1.68) and DFS/PFS/RFS (HR = 1.75) (Supplementary Table S2; Figure 6) (42, 43).

#### Bladder cancer (BC)

Bladder cancer patients with low PNI experienced decreased OS (HR = 1.80) and RFS (HR = 1.53) (Supplementary Table S2; Figure 6) (44).

#### Urinary malignancies (UM)

Uveal melanoma (UM) patients with low PNI had poorer OS (HR = 1.72) (Supplementary Table S2; Figure 6) (45).

### Outcomes of lung cancer

#### Small cell lung cancer (SCLC)

**Figure 7:**
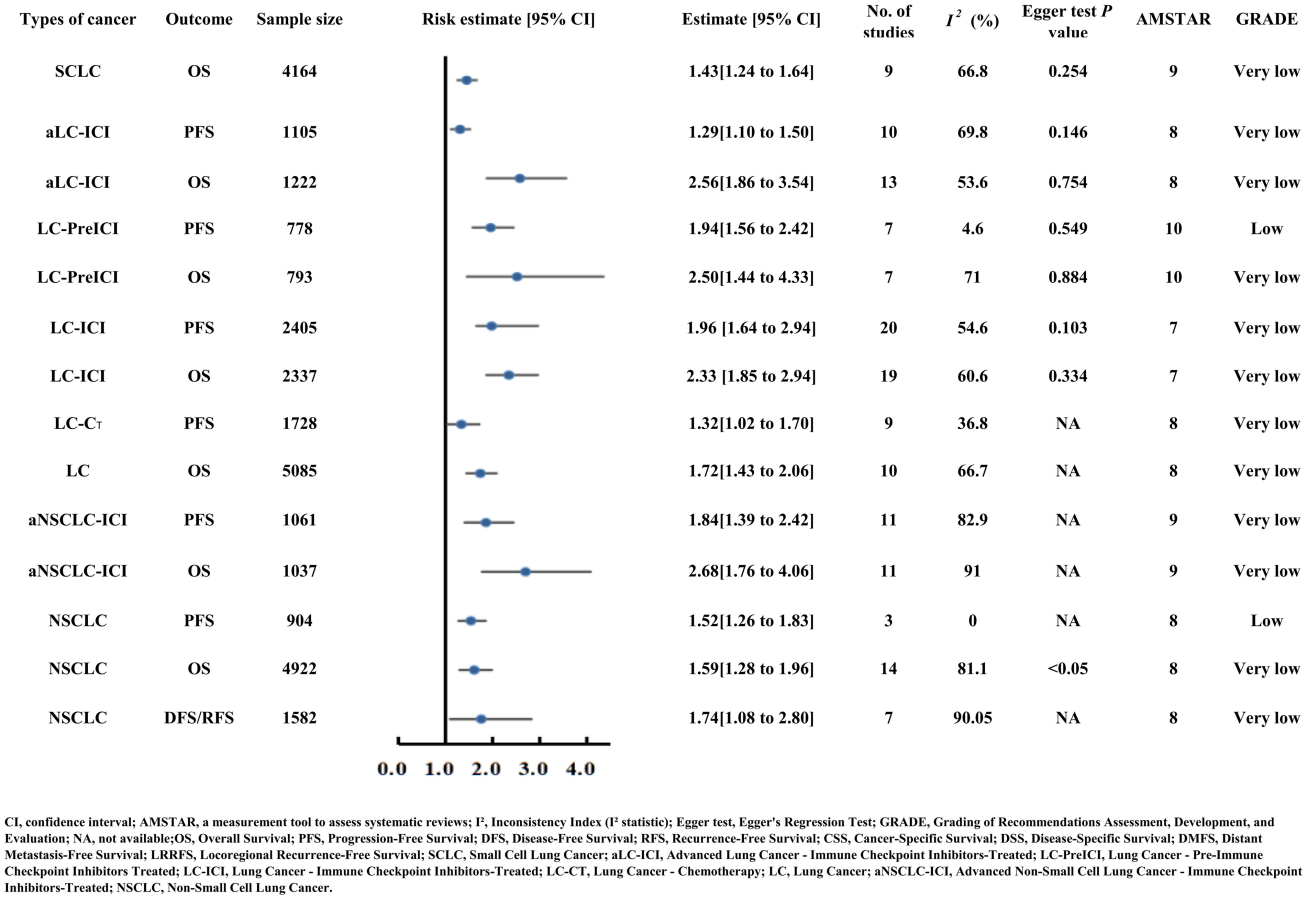
Forest plot illustrating risk estimates and 95% confidence intervals for lung cancer (SCLC, NSCLC, advanced lung cancer) and outcomes such as OS and PFS, including subgroups receiving immunotherapy, chemotherapy, or pre-immunotherapy. Key metrics include sample size, study count, heterogeneity, Egger test results, AMSTAR scores, and GRADE quality (low to very low).

Small cell lung cancer (SCLC) patients with low PNI faced poorer OS (HR = 1.43) (Supplementary Table S2; Figure 7) (46).

#### Non-small cell lung cancer (NSCLC)

Non-small cell lung cancer (NSCLC) patients demonstrated compromised OS (HR = 1.59), PFS (HR = 1.52), and DFS/RFS (HR = 1.74) with low PNI. Advanced NSCLC patients receiving immune checkpoint inhibitors (aNSCLC-ICI) had worse OS (HR = 2.68) and PFS (HR = 1.84) when PNI was reduced (Supplementary Table S2; Figure 7) (47, 48).

#### Overall lung cancer

In lung cancer patients, a low PNI was associated with poorer OS (HR = 1.72) (Supplementary Table S2; Figure 7) (49). Specifically, for those receiving immune checkpoint inhibitor therapy, both the advanced cohort (aLC-ICI) and the broader LC-ICI cohort faced increased OS risk (aLC-ICI HR = 2.56; LC-ICI HR = 2.33) and PFS risk (aLC-ICI HR = 1.29; LC-ICI HR = 1.96) with low PNI (Supplementary Table S2; Figure 7) (50, 51). Notably, prior to immunotherapy, patients with low PNI had a 150% increased OS risk (HR = 2.50), and 94% increased PFS risk (HR = 1.94) (Supplementary Table S2; Figure 7) (52). Separately, among lung cancer patients receiving chemotherapy (LC-CT), low PNI was linked to poorer PFS (HR = 1.32) (Supplementary Table S2; Figure 7) (53).

### Outcomes of head and neck cancer (HNC)

#### Head and neck cancer

**Figure 8:**
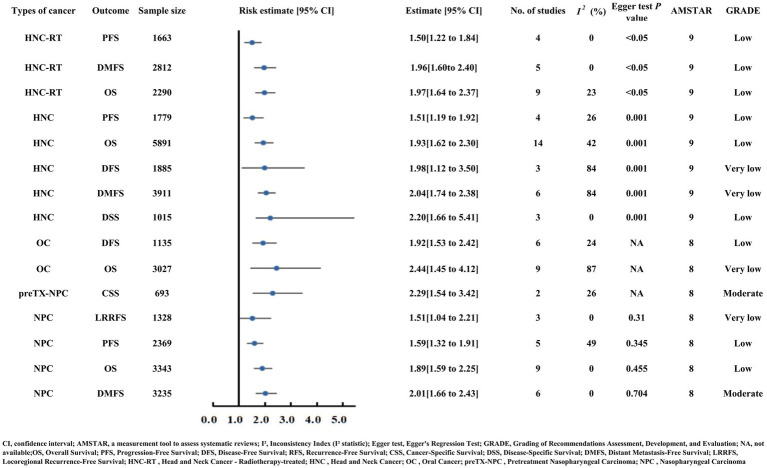
Forest plot showing risk estimates and 95% confidence intervals for head and neck cancer, oral cancer, and nasopharyngeal carcinoma, with outcomes including OS, PFS, DFS, DMFS, and CSS. It presents sample size, number of studies, I² inconsistency, Egger test P values, AMSTAR scores, and GRADE levels.

Head and neck cancer (HNC) patients with low PNI had poorer OS (HR = 1.93), DMFS (HR = 2.04), and DSS (HR = 2.20) (Supplementary Table S2; Figure 8) (54). Similarly, patients undergoing radiotherapy (HNC-RT) demonstrated poorer DMFS (HR = 1.96) and OS (HR = 1.97) with low PNI (Supplementary Table S2; Figure 8) (55).

#### Nasopharyngeal carcinoma (NPC)

Nasopharyngeal carcinoma (NPC) patients with low PNI showed poorer OS (HR = 1.89) and PFS (HR = 1.59) (Supplementary Table S2; Figure 8) (56). Moderate-quality evidence highlighted two key findings: low PNI correlated with worse DMFS (HR = 2.01) and pretreatment low PNI elevated CSS risk (HR = 2.29, 129% increased risk) with consistent results and no publication bias (Egger’s test *p* > 0.05) (57).

#### Oral cancer (OC)

In the context of oral cancer, patients with low PNI had poorer OS (HR = 2.44) and DFS (HR = 1.92) (Supplementary Table S2; Figure 8) (58).

### Outcomes of central nervous system malignancies

**Figure 9:**
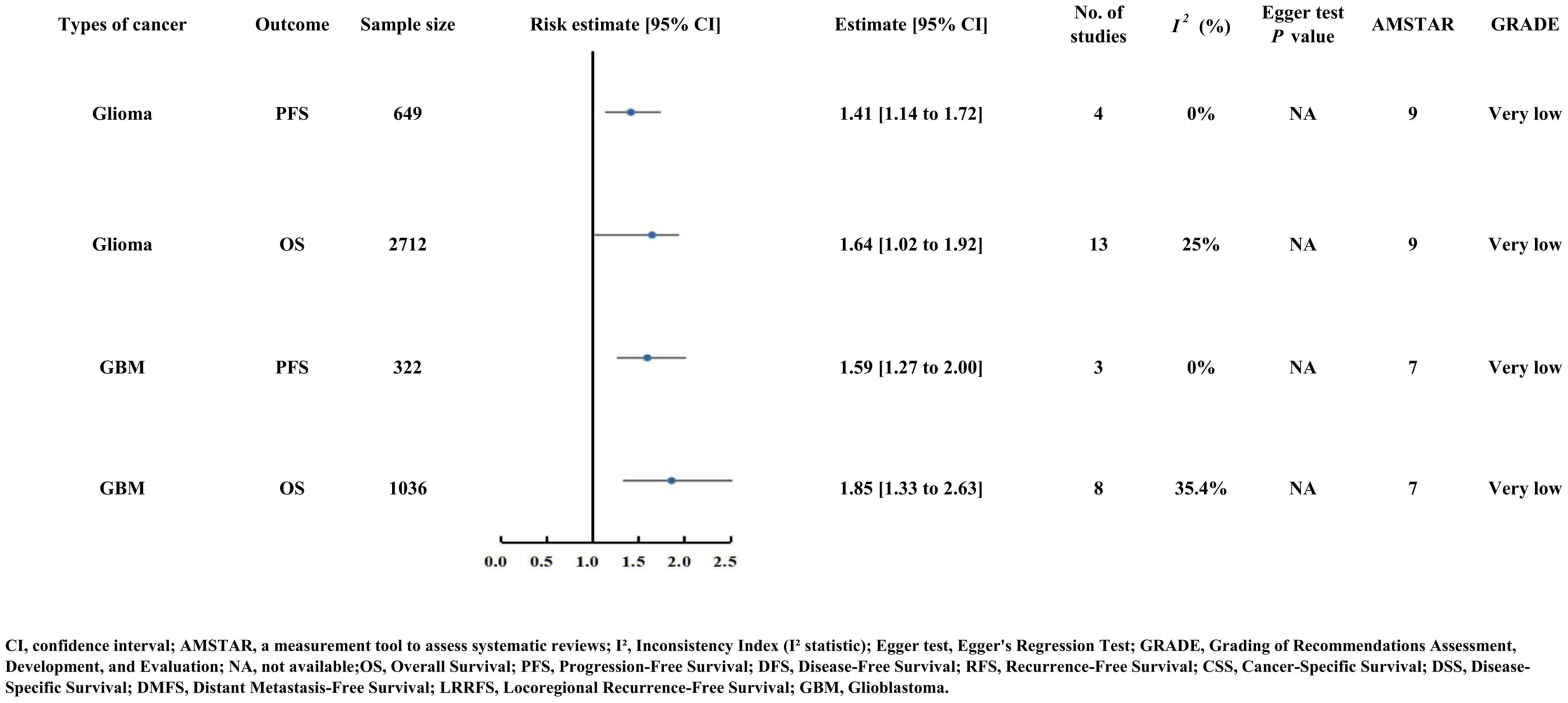
Forest plot of risk estimates and 95% confidence intervals for glioma and glioblastoma (GBM) with outcomes of OS and PFS. Details include sample size, number of studies, heterogeneity (I²), Egger test P values, AMSTAR scores, and GRADE quality (very low).

Glioma patients with low PNI had elevated PFS (HR = 1.41) and OS (HR = 1.64) risk. Glioblastoma (GBM) patients also showed increased PFS (HR = 1.59) and OS (HR = 1.85) risk with low PNI (Supplementary Table S2; Figure 9) (59, 60). All analyses were graded as Very low certainty evidence (GRADE), and publication bias could not be assessed due to insufficient studies (Egger’s test NA).

### Outcomes of lymphoma

**Figure 10:**
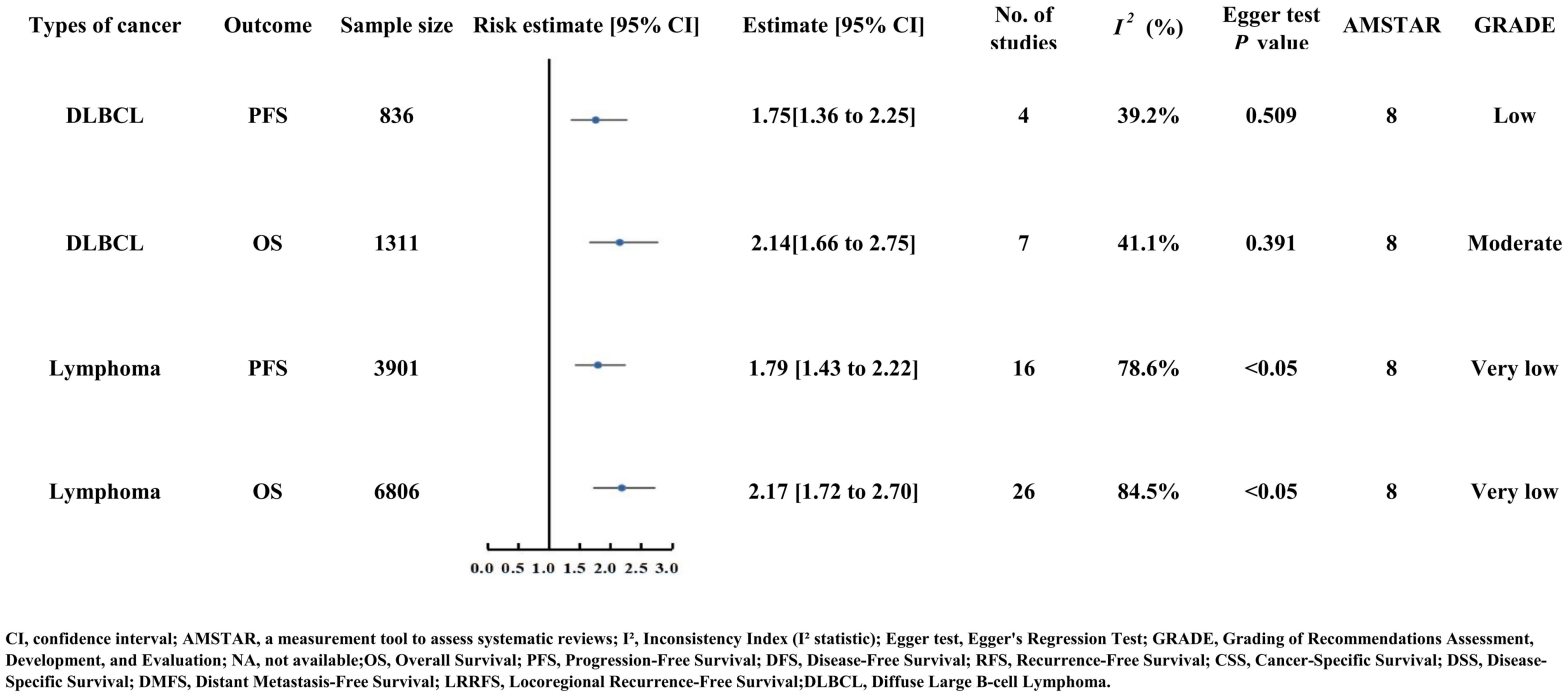
Forest plot displaying risk estimates and 95% confidence intervals for lymphoma, particularly diffuse large B-cell lymphoma (DLBCL), with outcomes of OS and PFS. Key data include sample size, number of studies, heterogeneity (I²), Egger test P values, AMSTAR scores (8), and GRADE quality (low to moderate).

Overall, lymphoma patients with low PNI faced significantly elevated OS (HR = 2.17) and PFS (HR = 1.79) risk (Supplementary Table S2; Figure 10). Diffuse large B-cell lymphoma (DLBCL) provided moderate-quality evidence: low PNI predicted increased OS risk (HR = 2.14, 114% increase) based on seven studies (*n* = 1,311) with moderate heterogeneity (I^2^ = 41.1%) and no publication bias (Egger’s test *p* = 0.391). For PFS, low PNI was associated with a significant progression risk (HR = 1.75) (Figure 10) (61, 62).

### Outcomes of other unclassified tumors

#### Unclassified overall cancer patients

**Figure 11:**
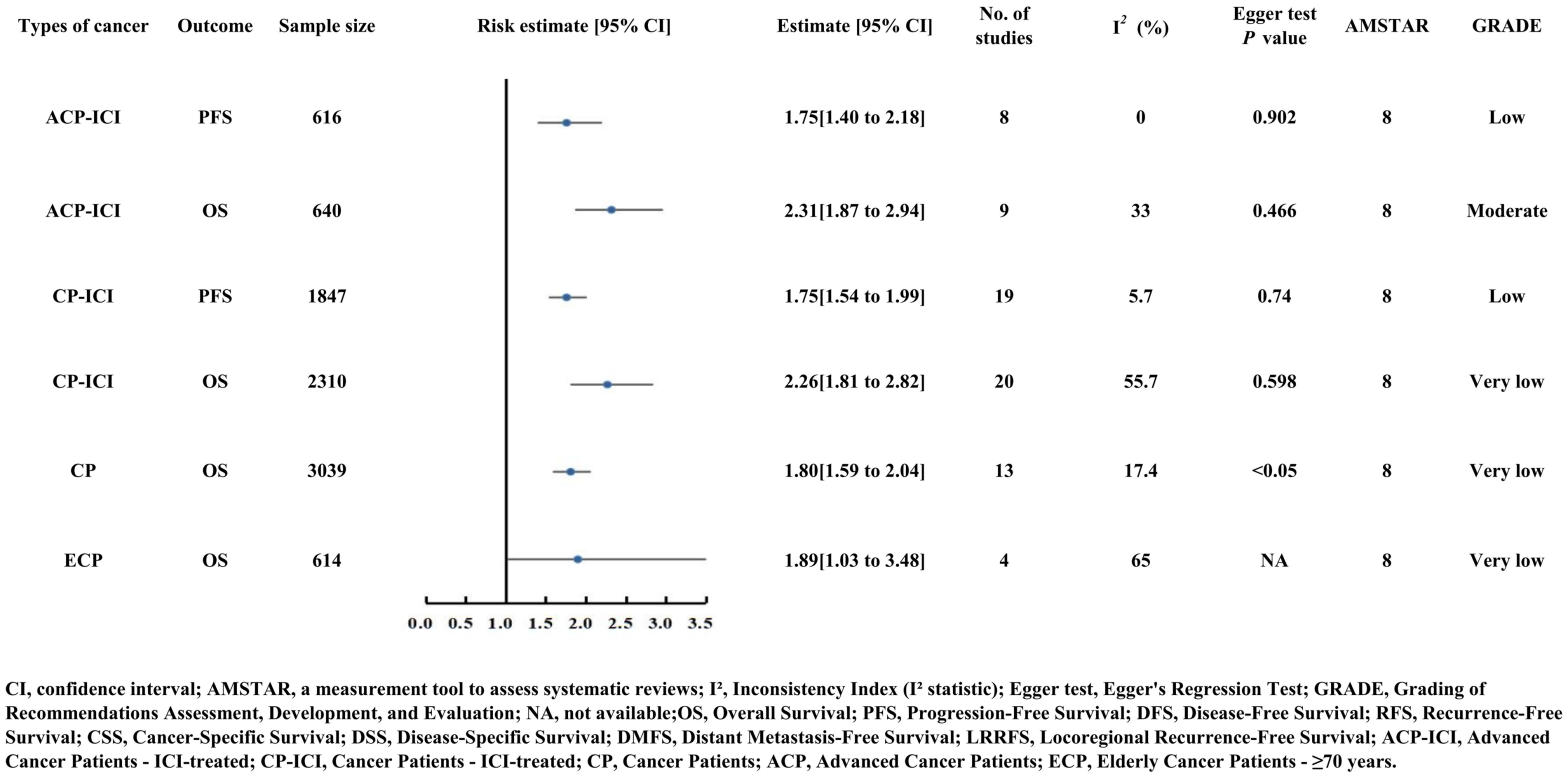
Forest plot of risk estimates and 95% confidence intervals for three subgroups of unclassified tumors: unclassified overall cancer patients, elderly cancer patients (age ≥70 years), and cancer patients treated with immune checkpoint inhibitors (CP-ICI, ACP-ICI). Outcomes focus on OS and PFS, with key metrics including sample size, number of studies, heterogeneity (I²), Egger test P values, AMSTAR scores, and GRADE levels. Moderate-quality evidence is noted for ACP-ICI OS (HR=2.31).

Unclassified cancer patients with low PNI had shortened OS (HR = 1.80) (Supplementary Table S2; Figure 11) (63).

#### Elderly cancer patients (Age ≥ 70 Years)

Elderly cancer patients (age ≥ 70 years) with low PNI had increased OS risk (HR = 1.89) (Supplementary Table S2; Figure 11) (64).

#### Cancer patients treated with immune checkpoint Inhibitor

Cancer patients receiving immune checkpoint inhibitors (CP-ICI and ACP-ICI) had poorer OS (CP-ICI HR = 2.26; ACP-ICI HR = 2.31) and PFS (both HR = 1.75) with low PNI (Supplementary Table S2; Figure 11) (65, 66). Notably, moderate-quality evidence emerged for ACP-ICI patients: low PNI was linked to a 131% increased OS risk (HR = 2.31) based on nine studies (*n* = 640) with low heterogeneity (I^2^ = 33%) and no publication bias (Egger’s test *p* = 0.466) (Figure 11) (66).

### Heterogeneity

In this study, a total of 111 heterogeneity indicators (I^2^) were extracted, among which 53 (47.8%) demonstrated significant heterogeneity (I^2^ > 50%). It is noteworthy that most outcome indicators with non-significant HR values exhibited significant heterogeneity. The potential sources of this heterogeneity may be attributed to variations in cancer types and stages, differences in patient baseline characteristics, discrepancies in treatment regimens, and methodological differences across the studies (Supplementary Tables S2, S3).

### Egger test

A total of 72 Egger test results were analyzed, with 29 (40%) indicating significant publication bias (*p* < 0.05). For studies lacking extracted Egger test results, publication bias was evaluated through the examination of funnel plots, and these findings were integrated into the GRADE rating assessment. Notably, 15 studies within the included literature did not evaluate publication bias (Supplementary Tables S1–S3).

### AMSTAR scores and GRADE ratings

The AMSTAR scores were predominantly high, with 92.7% of studies achieving scores of ≥ 8 points, thereby indicating a robust methodological quality in the majority of the meta-analyses. GRADE ratings were Moderate (medium) (11 studies), Low (low) (34 studies), Very low (very low) (66 studies).

The following 11 studies were rated as having moderate-quality evidence: Breast Cancer (Pre-treatment)-OS (32), GIST-RFS (30), Diffuse Large B-cell Lymphoma-OS (62), Advanced Cancer (Treated with Immune Checkpoint Inhibitors)-OS (66), Breast Cancer (Post-surgery)-OS (33), Cervical Cancer-PFS (38), Nasopharyngeal Carcinoma-DMFS (56), Cervical Cancer-OS (38), Esophageal Cancer-CSS (22), Nasopharyngeal Carcinoma (Pre-treatment)-CSS (57), and Renal Cancer (Advanced Targeted Therapy)-PFS (Supplementary Tables S1–S3) (40).

## Discussion

The study determined that a low Prognostic Nutritional Index (PNI) is significantly correlated with poor prognosis across nearly all tumor types, encompassing 12 categories of malignancies, including those of the digestive, reproductive, and urinary systems. This widespread association implies that PNI may represent a common pathological mechanism in cancer progression, particularly reflecting an imbalance in the nutrition-immunity-inflammation axis. Among the 74 meta-analyses included, the HR for 103 significantly associated outcomes predominantly ranged from 1.5 to 3.0, demonstrating the stable prognostic predictive capacity of PNI.

In the context of breast cancer, both preoperative and postoperative Prognostic Nutritional Index (PNI) demonstrated OS HR exceeding 2.5, with values of 2.56 and 2.63, respectively, and the quality of evidence was assessed as moderate (32, 33). The influence of PNI on OS was particularly significant in cervical cancer, with an HR of 2.98, while maintaining low heterogeneity (I^2^ = 6.1%) (38). Subgroups undergoing immunotherapy exhibited consistently stronger associations between PNI and OS compared to those receiving conventional treatments, with this effect being especially pronounced in patients with lung cancer. Specifically, the effect sizes of PNI in lung cancer immunotherapy cohorts (HR ranging from 2.56 to 2.68) exceeded those observed in both other cancer immunotherapy groups and conventional lung cancer treatment groups (e.g., lung cancer immunotherapy cohort OS HR = 2.33 [95% CI: 1.85 to 2.94] versus conventional lung cancer cohort OS HR = 1.72 [95%CI: 1.43 to 2.06]) (49, 51). This disparity may reflect the unique reliance of immunotherapy on the nutritional-immune status of patients, particularly in highly immunogenic tumors such as lung cancer.

Among studies of moderate quality, all 11 pieces of evidence demonstrated large effect sizes. These robust associations were primarily observed in the contexts of OS for breast cancer, RFS for gastrointestinal stromal tumors, OS for diffuse large B-cell lymphoma, OS for patients with advanced diseases treated with immunotherapy, DMFS for nasopharyngeal carcinoma, and both OS and PFS for cervical cancer (30, 32, 33, 38, 56, 62, 66). This consistency indicates that with a rigorous study design (AMSTAR score ≥ 8) and controlled heterogeneity (I^2^ < 50%), the true effect of PNI may be more substantial than previously recognized. These findings underscore the potential clinical applications of PNI in preoperative risk assessment for breast and cervical cancers, in predicting the efficacy of immunotherapy in patients, and in monitoring recurrence for gastrointestinal stromal tumors.

For the evidence rated as “Moderate certainty” (such as OS in breast cancer and cervical cancer), it means we have “moderate confidence” that the true effect is close to the estimated effect. These findings can provide valuable references for specific clinical scenarios (e.g., preoperative risk assessment), but future studies may still modify this estimate. It is important to emphasize that this is not conclusive evidence and cannot be directly used as an independent clinical decision-making tool, thus avoiding overinterpretation. This helps clinicians more accurately understand the applicable boundaries of the study results.

Malnutrition is a common condition among cancer patients and has a profound effect on their survival outcomes. The nutritional status of these patients is closely linked to their overall health and plays a crucial role in tumor development, progression, and response to treatment. Research indicates that malnutrition results in compromised immune function, decreased tolerance to treatment, and heightened postoperative complications, all of which contribute to reduced survival rates (67–70). Therefore, evaluating the nutritional status of cancer patients is essential for optimizing treatment strategies and enhancing survival outcomes.

The PNI serves as an indicator of patients’ nutritional and immune status by incorporating serum albumin and peripheral lymphocyte counts, and it has demonstrated significant prognostic value across various cancer types. The Controlling Nutritional Status (CONUT) score combines serum albumin, lymphocyte count, and cholesterol levels to provide a comprehensive evaluation of nutritional status. Consequently, there is a notable inverse correlation between PNI and CONUT, with low PNI values often coinciding with high CONUT scores. The Geriatric Nutritional Risk Index (GNRI) is primarily used to assess nutritional risk in elderly patients and has been shown to have prognostic relevance in several malignancies (71).

In the nutritional assessment of cancer patients, PNI, CONUT, and GNRI are three commonly employed metrics. For instance, research by Xiao et al. (72) has demonstrated that PNI acts as an independent prognostic marker in esophageal cancer, effectively stratifying patients based on survival outcomes. Similarly, Xie et al. (73) have reported that elevated CONUT scores are predictive of poor prognosis in patients with NSCLC. Furthermore, Zhang et al. (74) have identified GNRI as a valuable predictor of long-term survival and postoperative complications in gastric cancer patients. Zhang et al. (4) found that malnutrition remains prevalent among elderly cancer patients regardless of assessment tool, correlating with reduced quality of life and diminished immunotherapy response.

Conflicting reports continue to emerge regarding the prognostic significance of the PNI, the CONUT score, and the Geriatric Nutritional Risk Index (GNRI) across various tumor types. For instance, Zheng et al. (75) demonstrated through multivariable analysis that the CONUT score independently predicts OS and CSS in RCC patients undergoing nephrectomy, whereas PNI and three other factors do not. Similarly, Zhang et al. (15) and Kim et al. (76) identified the CONUT score as an independent prognostic marker for poor prognosis in stage III-IV NSCLC, surpassing both the systemic inflammatory index (SII) and PNI. Liu et al. (77) found that the CONUT score identifies patients with poor prognosis who are not detected by PNI, potentially due to unmeasured cholesterol concentrations in PNI, suggesting that CONUT may offer a more comprehensive nutritional risk assessment in gastric cancer. Conversely, Kim et al. (74, 76) established that PNI serves as a more significant preoperative predictor of OS than CONUT or inflammatory biomarkers in stage I-III colorectal cancer following surgical resection. Huang et al. (78) demonstrated that a low GNRI predicts poor OS and PFS in HNC patients, while CONUT did not predict outcomes after adjusting for confounding factors. Notably, Peng et al. (12) confirmed that both CONUT and PNI effectively predict OS in breast cancer, although their findings align with our pooled analysis.

**Figure 12:**
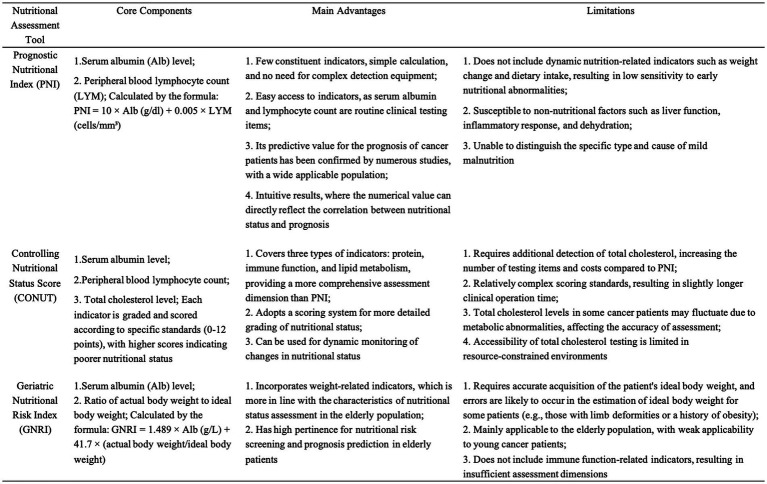
Comparative table of three nutritional assessment tools (Prognostic Nutritional Index [PNI], Controlling Nutritional Status Score [CONUT], Geriatric Nutritional Risk Index [GNRI]) detailing their core components, main advantages, and limitations.

The PNI demonstrates notable limitations as an index for assessing nutritional and immune status, primarily due to its low sensitivity to variations in body weight and dietary intake. This limitation is compounded by its reliance on only two parameters: serum albumin and lymphocyte count, which fail to provide a comprehensive reflection of a patient’s nutritional status. Nevertheless, the simplicity of PNI—with its reliance on two readily available, routine clinical parameters—renders it practically advantageous in clinical settings, particularly in resource-constrained environments where complex assessment tools may be inaccessible. Its ease of calculation further enhances its utility for rapid nutritional screening in busy clinical workflows. Conversely, the CONUT score incorporates three parameters—serum albumin, lymphocyte count, and total cholesterol levels—thereby offering a more multidimensional and comprehensive nutritional assessment. Nonetheless, the relatively complex calculation involved in CONUT poses challenges for clinical application (79). Additionally, cholesterol measurements may be subject to variability due to differing laboratory methodologies and individual variations in lipid metabolism, which could affect the accuracy and reliability of the assessment. The GNRI, specifically designed for older adults, utilizes two critical parameters: body mass index and serum albumin levels. By incorporating the ideal weight ratio (actual weight/ideal weight), GNRI effectively identifies abnormal weight changes prevalent in elderly populations. The inclusion of serum albumin further facilitates the concurrent evaluation of nutritional risk, inflammatory status, and the severity of protein-energy malnutrition, which is particularly crucial for this demographic (80). To provide a clear overview of the components, advantages, and limitations of these three nutritional assessment tools, a summary is presented in Figure 12.

The present research demonstrates considerable limitations in terms of both the quantity of studies and the quality of evidence, thereby hindering definitive conclusions about the clinical efficacy and applicability of the PNI, CONUT, and GNRI nutritional assessment indices. Importantly, this study constitutes the inaugural systematic evaluation of the associations between PNI and survival outcomes across various malignancies through the use of umbrella meta-analysis, a methodology that facilitates comprehensive evidence integration and assessment of reliability. However, even this sophisticated evidence synthesis approach is constrained by deficiencies in the primary studies, including publication bias, methodological heterogeneity, and incomplete data reporting, which may undermine the robustness and generalizability of the conclusions.

The reduction of the Prognostic Nutritional Index (PNI) mainly affects tumors through three key mechanisms. First, it directly suppresses anti-tumor immune function: Hypoalbuminemia limits the supply of amino acids and carrier proteins required for lymphocyte proliferation, leading to reduced CD8 + T cell cytotoxicity, impaired helper T cell-mediated cytokine secretion (e.g., IL-2 and IFN-*γ*), and decreased NK cell activity, all of which compromise immune surveillance against tumors (81). Second, it modulates the tumor inflammatory microenvironment: Malnutrition associated with low PNI induces chronic inflammation, characterized by upregulated IL-6 and TNF-*α*. These cytokines activate the NF-κB signaling pathway, thereby promoting tumor proliferation, angiogenesis, and metastasis (82). Additionally, hypoalbuminemia-induced reduction in circulating essential amino acids such as glutamine may indirectly contribute to the immunosuppressive state of the tumor microenvironment (82–84).

Third, it impairs treatment tolerance: Hypoalbuminemia weakens the intestinal mucosal barrier, increasing the risk of bacterial translocation and severe complications (e.g., mucositis and diarrhea) during chemotherapy with drugs like 5-fluorouracil and methotrexate (85, 86). For chemotherapeutic agents relying on albumin binding (e.g., paclitaxel and irinotecan), Hypoalbuminemia raises unbound drug concentrations, accelerating clearance, enhancing toxicity (e.g., bone marrow suppression and neurotoxicity), and potentially reducing effective drug levels in tumors, which may compromise therapeutic efficacy (87). Collectively, these mechanisms underscore the critical role of PNI in mediating the crosstalk between nutritional status, immune function, and tumor progression, providing a biological basis for its prognostic value across diverse cancer types.

### Strengths

This study conducted an umbrella review integrating 74 meta-analyses, encompassing 159 outcome measures across 12 categories of malignancies, including those of the digestive, reproductive, urinary, and respiratory systems, among others. It systematically assessed, for the first time, the prognostic value of the PNI across various tumor types. This extensive synthesis of evidence addresses the limitations inherent in individual meta-analyses or original studies, thereby providing more robust evidence-based support for clinical practice. The study adhered rigorously to systematic methodologies, following the PRISMA and SIGN guidelines, with dual independent retrieval and screening processes, and arbitration by a third researcher to minimize selection bias. We reanalyzed hazard ratios and odds ratios using random-effects or fixed-effect models, calculating 95% confidence intervals, and assessed heterogeneity and publication bias for each included meta-analysis. Furthermore, the quality of evidence and methodological rigor for each survival outcome were evaluated using GRADE grading and AMSTAR scoring, and we assessed our confidence in these estimates. The study elucidated the widespread association of PNI with various malignancies.

### Limitations

It is important to emphasize that the unit of analysis in this study was the “specific patient population–specific outcome” combination. Therefore, even if two meta-analyses both focused on “renal cancer,” they were considered independent analyses addressing distinct clinical questions and were both included—for instance, if one examined OS in “surgical patients” and the other focused on PFS in “targeted therapy patients.” However, potential overlap of patient cohorts across different subgroup analyses within the same meta-analysis—for example, between an overall population analysis and one of its subgroups—was not identified or adjusted for, which may introduce some imprecision in the pooled effect estimates.

A key finding of our study lies in the systematic evaluation of the existing evidence quality. Although our analysis indicates that a low PNI is broadly associated with poorer survival outcomes across multiple cancer types, it must be emphasized that the majority of evidence supporting these associations (89.3%) was rated as “Low” or “Very low” certainty according to the GRADE framework. This judgment primarily stems from potential risks of bias in the original studies, widespread high heterogeneity among studies (as indicated by high I^2^ values), and potential publication bias. Therefore, we strongly recommend that the effect estimates (e.g., hazard ratio and HR) for most associations reported in this study should be interpreted with extreme caution. These findings should be regarded as robust clues for “hypothesis generation” and “guiding future research directions” rather than as solid evidence to directly influence current clinical decision-making. There is an urgent need for more rigorously designed, prospective, multicenter studies to validate these findings, particularly in specific cancer types where Moderate certainty evidence has already been identified. Furthermore, most existing studies focused on baseline PNI, lacking data on the relationship between dynamic monitoring of PNI during treatment and prognosis. This limitation hinders the exploration of the timing and effectiveness of nutritional interventions. Additionally, although CONUT and GNRI were discussed, the predictive performance of these three indicators was not evaluated through direct statistical comparisons, such as network meta-analysis, thereby complicating the ability to clearly recommend a preferred indicator.

## Conclusion

A low Prognostic Nutritional Index is consistently associated with poorer survival outcomes across a range of cancer types. This association is supported by Moderate-certainty evidence in several specific clinical contexts, including overall survival in breast cancer, overall and progression-free survival in cervical cancer, relapse-free survival in gastrointestinal stromal tumors, overall survival in diffuse large B-cell lymphoma, and selected advanced cancers treated with immune checkpoint inhibitors. For the majority of other cancer types and outcomes, however, the supporting evidence remains of Low or Very low certainty, primarily due to substantial heterogeneity and potential publication bias. Consequently, despite its consistent prognostic value, the current evidence is insufficient to support the routine use of PNI as a standalone prognostic or decision-making tool across all tumor types. Future rigorously designed, multi-center studies are warranted to establish its clinical utility in well-defined populations and settings.

## Data Availability

The original contributions presented in the study are included in the article/Supplementary material, further inquiries can be directed to the corresponding authors.
